# Molecular characterisation of the tick *Rhipicephalus microplus* in Malaysia: new insights into the cryptic diversity and distinct genetic assemblages throughout the world

**DOI:** 10.1186/s13071-015-0956-5

**Published:** 2015-06-24

**Authors:** Van Lun Low, Sun Tee Tay, Kai Ling Kho, Fui Xian Koh, Tiong Kai Tan, Yvonne Ai Lian Lim, Bee Lee Ong, Chandrawathani Panchadcharam, Yusoff Norma-Rashid, Mohd Sofian-Azirun

**Affiliations:** Institute of Biological Sciences, Faculty of Science, University of Malaya, Kuala Lumpur, Malaysia; Department of Medical Microbiology, Faculty of Medicine, University of Malaya, Kuala Lumpur, Malaysia; Department of Parasitology, Faculty of Medicine, University of Malaya, Kuala Lumpur, Malaysia; Faculty of Veterinary Medicine, Universiti Malaysia Kelantan, Kota Bharu, Kelantan Malaysia; Veterinary Research Institute, Ipoh, Perak Malaysia

**Keywords:** *Rhipicephalus microplus*, Cattle Tick, COI Gene, 16S rRNA Gene, Malaysia

## Abstract

**Background:**

The morphotaxonomy of *Rhipicephalus microplus* complex has been challenged in the last few years and prompted many biologists to adopt a DNA-based method for distinguishing the members of this group. In the present study, we used a mitochondrial DNA analysis to characterise the genetic assemblages, population structure and dispersal pattern of *R. microplus* from Southeast Asia, the region where the species originated.

**Methods:**

A phylogeographic analysis inferred from the 16S rRNA and cytochrome oxidase subunit I (COI) genes was performed with five populations of *R. microplus* collected from cattle in Malaysia. Malaysian *R. microplus* sequences were compared with existing COI and 16S rRNA haplotypes reported globally in NCBI GenBank.

**Results:**

A total of seven and 12 unique haplotypes were recovered by the 16S rRNA and COI genes, respectively. The concatenated sequences of both 16S rRNA and COI revealed 18 haplotypes. Haplotype network and phylogenetic analyses based on COI+16S rRNA sequences revealed four genetically divergent groups among Malaysian *R. microplus*. The significantly low genetic differentiation and high gene flow among Malaysian *R. microplus* populations supports the occurrence of genetic admixture. In a broader context, the 16S rRNA phylogenetic tree assigned all isolates of Malaysian *R. microplus* into the previously described African/the Americas assemblage. However, the COI phylogenetic tree provides higher resolution of *R. microplus* with the identification of three main assemblages: clade A sensu Burger *et al.* (2014) comprises ticks from Southeast Asia, the Americas and China; clade B sensu Burger *et al.* (2014) is restricted to ticks that originated from China; and clade C sensu Low *et al.* (2015) is a new genetic assemblage discovered in this study comprising ticks from India and Malaysia.

**Conclusions:**

We conclude that the *R. microplus* complex consisting of at least five taxa: *R. australis*, *R. annulatus*, *R. microplus* clade A sensu Burger *et al.* (2014), *R. microplus* clade B sensu Burger *et al.* (2014) and the new taxon, *R. microplus* clade C sensu Low *et al.* (2015). The use of COI as the standard genetic marker in discerning the genetic assemblages of *R. microplus* from a broad range of biogeographical regions is proposed.

**Electronic supplementary material:**

The online version of this article (doi:10.1186/s13071-015-0956-5) contains supplementary material, which is available to authorized users.

## Background

The southern cattle tick, *Rhipicephalus microplus* (formerly *Boophilus microplus*), is the most notorious blood-feeding ectoparasite of livestock, especially cattle. Over the years, the veterinary importance of *R. microplus* transmitting various pathogens has been acknowledged worldwide [1–7]. Additionally, the estimated annual losses associated with *R. microplus* are US$ 2.5 billion throughout tropical and subtropical regions [8].

To date, the ticks of the *R. microplus* complex consisting of four taxa, namely *R. australis*, *R. annulatus*, *R. microplus* clade A sensu Burger *et al.* (2014) and *R. microplus* clade B sensu Burger *et al.* (2014). Nonetheless, the morphotaxonomy of *R. microplus* complex has been challenged in the last few years and remained difficult to morphologically differentiate these members [9]. Molecular characterisation is the alternate way to distinguish these closely related taxa, as well as other *Rhipicephalus* ticks [10].

The phylogeography of *R. microplus* has been well-studied in many parts of the world using various molecular approaches, such as random amplified polymorphic DNA [11], restriction fragment length polymorphism [12] and microsatellite analyses [13, 14]. In 2009, Labruna *et al*. [15] used both mitochondrial genes (12S rRNA and 16S rRNA) and microsatellite markers to charactesize the genetic assemblages of *R. microplus* from Australia, Asia, Africa and the Americas. The results showed three distinct clades – one comprising ticks from the Americas and Africa; another comprising ticks from Australia, Indonesia and New Caledonia; and one comprising ticks from India and Nepal. As a result, *R. microplus* ticks from Australia, Indonesia and New Caledonia have been reinstated as *R. australis* based on reproductive isolation and taxonomic evidence [16]. A more recent study discovered a new mitochondrial COI gene lineage that is restricted to ticks from China, suggesting the presence of cryptic species that is more closely related to *R. annulatus* [10]. Thus far, the current literature has documented two distinct mitochondrial COI gene assemblages of *R. microplus*, so called clade A sensu Burger *et al.* (2014) and clade B sensu Burger *et al.* (2014) [9].

There is a gap in knowledge concerning the genetic assemblages and population structure of *R. microplus* from Southeast Asia. Previous studies have genetically analysed ticks from Australia, the Americas and Africa but sampling was limited, with most countries represented by a single individual. Small sample size provides little support for intraspecific genetic diversity and phylogenetic inferences [17]. Therefore, further studies on the population structure of Southeast Asian *R. microplus*, including those of Malaysia, are warranted. Given the high resolution of mitochondria-encoded 16S rRNA and COI genes reported in *Rhipicephalus* ticks [10, 15, 18], this study attempts to utilize these genes to reveal the hidden intraspecific genetic diversity and dispersal patterns of *R. microplus* for the first time in Malaysia. To infer the genetic assemblages of Malaysian *R. microplus*, sequences were compared with existing COI and 16S rRNA haplotypes reported globally in NCBI GenBank.

## Methods

### Ethics statement

All experiments were performed in accordance with relevant guidelines and regulations of the University of Malaya. The research protocols were regulated and approved by the University of Malaya. Prior to the commencement of the sample collection, permission was approved by the Department of Veterinary Services, Ministry of Agriculture and Agro-Based Industry, Malaysia (Reference Number: JPV/PSTT/100-8/1). This study did not involve endangered or protected species.

### Tick specimens

**Fig. 1:**
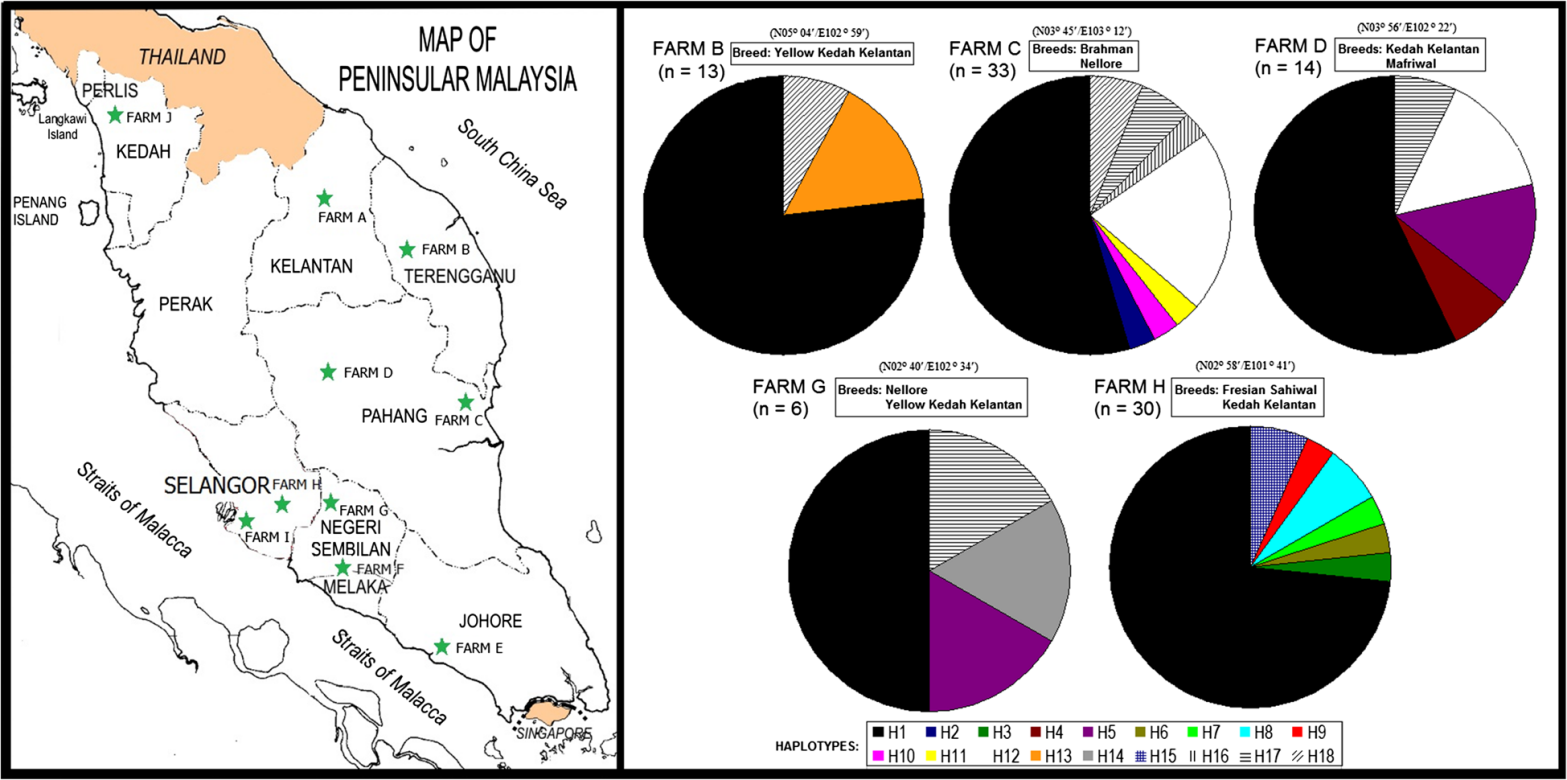
Collection sites of *R. microplus* and haplotype distribution (H1-H18) of COI + 16S rRNA sequences in Peninsular Malaysia

Tick collection was conducted on 10 animal farms (each farm with 30–40 individual animals) in seven states in Peninsular Malaysia, namely Kelantan (Farm A, Tanah Merah district), Terengganu (Farm B, Hulu Terengganu district), Pahang (Farm C, Kuantan district and Farm D, Jerantut district), Johore (Farm E, Batu Pahat district), Negeri Sembilan (Farm F, Jelebu district and Farm G, Gemas district), Selangor (Farm H, Serdang district and Farm I, Kuala Langat district) and Kedah (Farm J, Pokok Sena district) (Fig. 1). Cattle were raised at Farm A - Farm E and Farm G - Farm I, whereas sheep, goats and swine were raised at Farm J, Farm F and Farm I, respectively.

*Rhipicephalus microplus* was preliminary identified and separated from the closely related species *R. annulatus* by a set of morphological features: (1) short and deeply concave internal margin of the first palp article, (2) presence of a small spur on the second coxa of females, (3) presence of a caudal appendage in males [19]. To differentiate *R. microplus* from *R. australis*, the critical morphological features were: (1) dorsal setae are short and slender, and medial alloscutal setae form clusters of 2–3 rows in females, (2) absence of a spur on the ventral surface of first palp article of males, (3) presence of several setae on the lateral margins of the ventral surface of the capitulum in males [16]. The species identity was further confirmed by the 16S rRNA marker which can genetically separate the closely related species *R. annulatus* and *R. australis* [10, 15, 16].

*Rhipicephalus microplus* ticks were identified from cattle on five farms (Farms B, C, D, G and H) with low to high frequencies (personal observations). The cattle ticks were collected from three imported cattle breeds: Brahman (*n* = 3), Friesian Sahiwal (*n* = 4) and Nellore (*n* = 17); and three local cross breeds of cattle: Kedah Kelantan (*n* = 27), Mafriwal (*n* = 1) and Yellow Kedah Kelantan (*n* = 13). All *R. microplus* collected at Farm B (*n* = 13), Farm D (*n* = 14) and Farm G (*n* = 6) were used in DNA analyses, whereas randomly selected individuals from a larger sample from Farm C (*n* = 33, of 106) and Farm H (*n* = 30, of 249) were analysed. A total of 96 individual ticks from five farms were sequenced and analysed in this study (Table 1). The voucher specimens were deposited at the Museum of Zoology, University of Malaya, Malaysia.

**Table 1:** Sample sizes (N/n), haplotype diversity (Hd), nucleotide diversity (pi) and number of haplotypes (h) based on COI, 16S rRNA and COI + 16S rRNA sequences of *R. microplus* populations in Malaysia

Farm	N	n	COI			16S rRNA			COI + 16S rRNA		
			Hd	pi	h	Hd	Pi	h	Hd	pi	h
B	13	13	0.1539	0.0010	2	0.2821	0.0007	2	0.4103	0.0009	3 (H1,H13,H18)
C	106	33	0.6042	0.0023	5	0.1761	0.0005	4	0.6667	0.0016	8 (H1,H2,H10,H11,H12,H16,H17,H18)
D	14	14	0.6703	0.0296	5	0.0000	0.0000	1	0.6703	0.0016	5 (H1,H4,H5,H12,H17)
G	6	6	0.8000	0.0337	4	0.3333	0.0008	2	0.8000	0.0209	4 (H1,H5,H14,H17)
H	249	30	0.3632	0.0148	6	0.1862	0.0005	2	0.4644	0.0092	7 (H1,H3,H6,H7,H8,H9,H15)
Total	388	96	0.5123	0.0124	12	0.1787	0.0005	7	0.5875	0.0077	18 (H1-H18)

*N* = number of ticks collected; *n* = number of ticks genotyped

### Polymerase chain reaction (PCR)

*Rhipicephalus microplus* DNA was extracted from each specimen (*n* = 96), using QIAamp DNA Mini Kit (Qiagen, Hilden, Germany). The amplifications of the mitochondrial COI and 16S rRNA genes were performed in a final volume of 50 μL containing 50–100 ng genomic DNA, 25 μL of ExPrime Taq Master Mix (GENETBIO Inc., Daejeon, South Korea) and 10 pmol of each forward and reverse primer. PCR was conducted with an Applied Biosystems Veriti 96-Well Thermal Cycler (Applied Biosystems, Inc., Foster City, CA, USA).

Low amplification success rates (<50 %) were found from three pairs of COI primers [20–22], a common problem in the recovery of COI fragment from tick specimens [23]. To solve this issue, the COI of *R. microplus* was amplified using nested PCR in the present study. The first PCR was performed using the cycling parameters and primer pairs (cox1F and cox1R) from Chitimia *et al*. [22]. For the nested PCR amplification, 1 μL of the product from the first amplification was used with the primers C1-J-1718 and C1-N-2329 from Shao *et al*. [21] in a 50 μL reaction mixture. The modified PCR cycling parameters were: 94 °C for 5 min, 35 cycles of 94 °C for 30 s, 59 °C for 1 min and 72 °C for 1 min; and 72 °C for 10 min. For the 16S rRNA gene, PCR amplification was performed using the primer pairs 16S-F and 16S-R1 and PCR cycling parameters described in Lv *et al*. [20]. A negative control was included in each PCR run.

### DNA sequencing and data analyses

Purified PCR products were sent to a commercial company for DNA sequencing in forward and reverse directions. Sequence data were analysed and edited using Sequence Scanner 1.0 (Applied Biosystems, Foster City, CA, USA) and BioEdit 7.0.9.0 [24]. The partition homogeneity test was conducted using PAUP 4.0b10 [25]. No significant differences were found among separate gene regions (*P* = 0.88); hence data were concatenated for relevant analyses. The aligned COI and 16S rRNA sequences comprised 625 bp and 399 bp, respectively. The multiple sequences of both COI and 16S rRNA were concatenated to yield a total length of 1024 bp. Sequence alignment of *R. microplus* haplotypes (H1-H18) based on COI + 16S rRNA genes is shown in Additional file 1: Figure S1. Distinct haplotypes of COI (KM246866-KM246877) and 16S rRNA (KM246878-KM246884) were deposited in NCBI GenBank.

A median-joining analysis implemented in the program SplitsTree4 4.13.1 [26] was used for the intraspecific analysis of the evolutionary relationships among haplotypes. Uncorrected (p) pairwise genetic distances were calculated using PAUP 4.0B10 [25] to assess the genetic divergence of *R. microplus* in both COI and 16S rRNA genes.

To assess the level of genetic differentiation, gene flow and genetic differentiation tests implemented in the program DnaSP 5.0 [27] were performed. Haplotype diversity (Hd), nucleotide diversity (pi), number of haplotypes (h), genetic differentiation (F_ST_) and gene flow (Nm) values were determined. The levels of genetic differentiation are defined as *F*_*ST*_ > 0.25 (great differentiation), 0.15 to 0.25 (moderate differentiation) and *F*_*ST*_ < 0.05 (negligible differentiation) [28]. The levels of gene flow are defined as Nm > 1 (high gene flow), 0.25 to 0.99 (intermediate gene flow) and Nm < 0.25 (low gene flow) [29].

The distinct COI and 16S rRNA haplotypes identified in the Malaysian *R. microplus* ticks were aligned with all representative sequences of *Rhipicephalus* taxa available in NCBI GenBank. *Hyalomma detritum* was used as an outgroup for the construction of phylogenetic trees based on COI and 16S rRNA sequences. A neighbor-joining (NJ) phylogenetic tree [30] was plotted using MEGA5 [31]. The NJ bootstrap values were estimated using 1000 replicates with Kimura’s two-parameter model of substitution (K2P distance) [32]. Gaps and missing data were eliminated. A maximum likelihood (ML) analysis was run in PhyML 3.0 [33], using a HKY85 model with parameters estimated by the program.

## Results

### Haplotype and nucleotide analyses

**Fig. 2:**
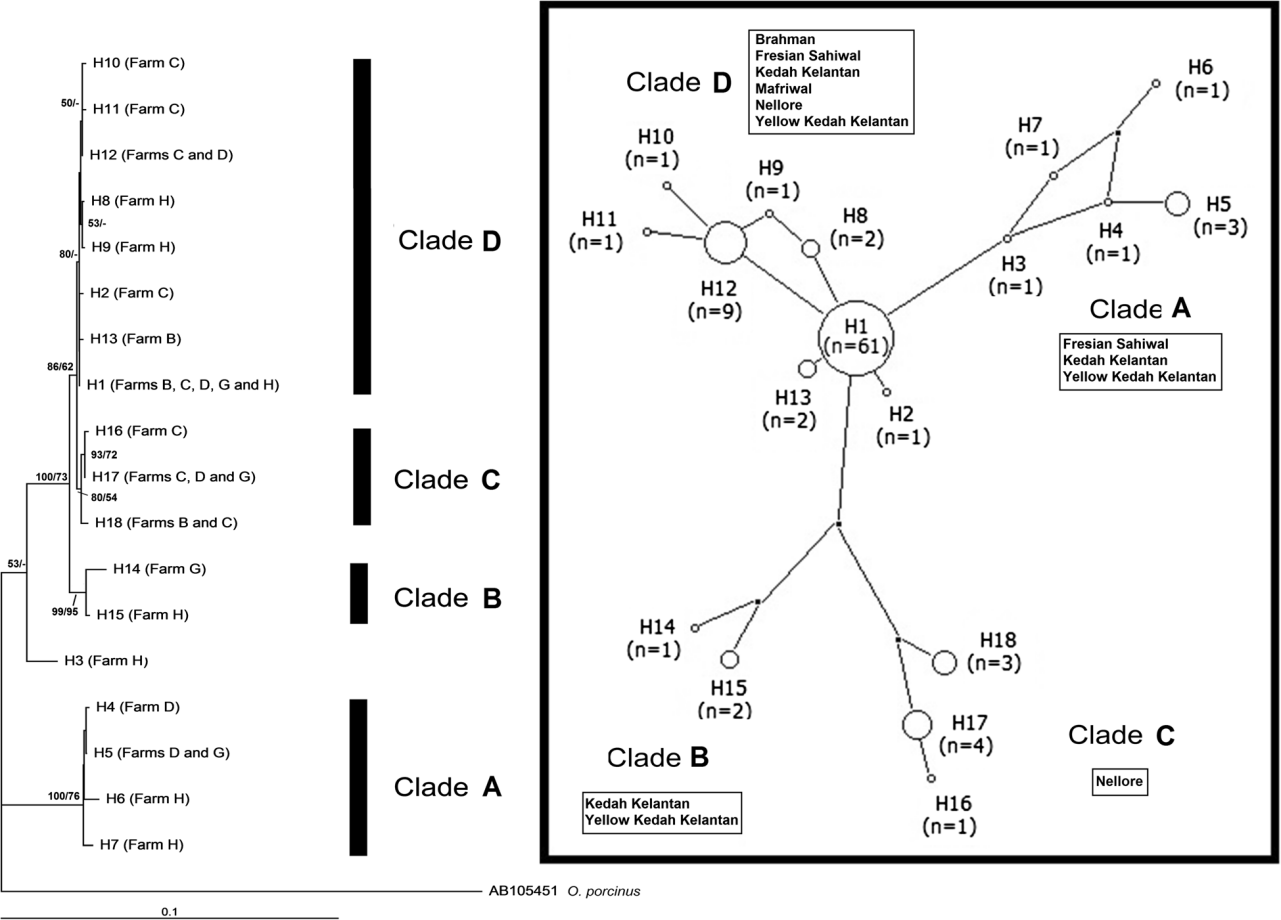
Neighbor-joining phylogenetic tree and median joining network of *R. microplus* from five different populations in Malaysia, based on combined COI + 16S rRNA sequences. Bootstrap values [neighbor-joining (NJ)/maximum likelihood (ML)] are shown on the branches. Each haplotype is represented by a circle. Relative sizes of the circles indicate haplotype frequency

A total of seven, 12 and 18 unique haplotypes were identified based on the sequence variation of the 16S rRNA, COI and COI + 16S rRNA genes, respectively, from 96 ticks collected from five farms in Peninsular Malaysia. The combined COI + 16S rRNA dataset showed a greater haplotype diversity (0.5875) than COI (0.5123) or 16S rRNA (0.1787) genes alone, whereas the COI gene showed greater nucleotide diversity (0.0124) than the combined COI + 16S rRNA (0.0077) dataset or 16S rRNA (0.0005) alone (Table 1). Haplotype network analysis (COI + 16S rRNA) showed four distinct haplotype clusters among Malaysian populations but revealed a lack of clear separation by tick populations/geographical areas, indicating an overlap of the four genetically divergent groups of *R. microplus*. Haplotype H1 was the most widespread haplotype (*n* = 61) in all populations (Figs. 1 and 2). Notably, the H16, H17 and H18 haplotypes, which mainly originated from the Nellore breed, formed a single cluster (clade C), shown in Fig. 2. However, the association of these haplotypes with other cattle breeds was not obvious.

### Genetic distance, genetic differentiation and gene flow

In the uncorrected “p” distance matrix, the COI gene indicated stronger resolving power (0.00–9.28 %) compared with 16S rRNA (0.00–0.50 %) (Table 2). The genetic distance of the representative COI + 16S rRNA haplotypes of *R. microplus* ranged from 0.10 to 5.76 % (Table 3). Overall, significant genetic differentiation was observed among all populations (*P* < 0.05). However, a relatively low level of genetic differentiation was found among the five tick populations. The majority of the population pairs showed low genetic differentiation. Moderate genetic differentiation (*F*_*ST*_ = 0.15), revealed by the COI and COI + 16S rRNA genes, was found between populations B and D. Additionally, high levels of gene flow were observed among tick populations as shown by the high Nm in the COI (36.92), 16S rRNA (12.47) and COI + 16S rRNA (35.59) genes (Table 4).

**Table 2:** Percentage of uncorrected “p” distance matrix between populations based on COI (upper right matrix) and 16S rRNA (lower left matrix) sequences of *R. microplus* in Malaysia

	B	C	D	G	H
B	-	0.00–0.80	0.00–8.48	0.00–8.32	0.00–8.64
C	0.00–0.50	-	0.00–8.64	0.00–8.48	0.00–9.12
D	0.00–0.25	0.00–0.25	-	0.00–8.80	0.00–8.96
G	0.00–0.50	0.00–0.50	0.00–0.25	-	0.00–9.28
H	0.00–0.50	0.00–0.50	0.00–0.25	0.00–0.50	-

**Table 3:** Percentage of uncorrected “p” distance matrix among the 18 representative COI + 16S rRNA haplotypes of *R. microplus* in Malaysia

	H1	H2	H3	H4	H5	H6	H7	H8	H9	H10	H11	H12	H13	H14	H15	H16	H17	H18
H1	-																	
H2	0.10	-																
H3	2.34	2.44	-															
H4	4.88	4.98	2.83	-														
H5	4.79	4.88	2.93	0.10	-													
H6	5.18	5.27	3.13	0.49	0.59	-												
H7	4.98	5.08	2.83	0.39	0.49	0.68	-											
H8	0.10	0.20	2.44	4.98	4.88	5.27	5.08	-										
H9	0.20	0.29	2.54	4.89	4.79	5.18	4.98	0.10	-									
H10	0.20	0.29	2.54	4.88	4.79	5.18	4.98	0.29	0.20	-								
H11	0.20	0.29	2.54	4.88	4.79	5.18	4.98	0.29	0.20	0.20	-							
H12	0.10	0.20	2.44	4.79	4.69	5.08	4.88	0.20	0.10	0.10	0.10	-						
H13	0.10	0.20	2.44	4.98	4.88	5.27	5.08	0.20	0.29	0.29	0.29	0.20	-					
H14	1.37	1.46	3.32	5.47	5.37	5.76	5.57	1.46	1.56	1.56	1.56	1.46	1.46	-				
H15	0.88	0.98	2.83	4.98	4.88	5.27	5.08	0.98	1.08	1.07	1.07	0.98	0.98	0.68	-			
H16	0.39	0.49	2.73	5.27	5.18	5.57	5.37	0.49	0.59	0.59	0.59	0.49	0.49	1.56	1.07	-		
H17	0.29	0.39	2.64	5.18	5.08	5.47	5.27	0.39	0.49	0.49	0.49	0.39	0.39	1.46	0.98	0.10	-	
H18	0.39	0.49	2.64	5.18	5.08	5.27	5.27	0.49	0.59	0.59	0.59	0.49	0.49	1.56	1.07	0.39	0.29	-

**Table 4:** Genetic differentiation (F_ST_) and gene flow (Nm) based on COI, 16S rRNA and COI + 16S rRNA sequences of *R. microplus* populations in Malaysia

Population 1	Population 2	COI	16S rRNA	COI + 16S rRNA
B	C	0.0474	0.0524	0.0483
B	D	0.1477	0.0833	0.1468
B	G	0.0220	0.0400	0.0226
B	H	0.0510	0.0777	0.0522
C	D	0.1344	0.0000	0.1334
C	G	0.0146	0.0000	0.0143
C	H	0.0565	0.0361	0.0558
D	G	−0.1067	0.0000	−0.1057
D	H	0.0091	0.0690	0.0095
G	H	−0.0778	0.0259	−0.0758
	Nm	36.92	12.47	35.59

### Phylogenetic analyses

**Fig. 3:**
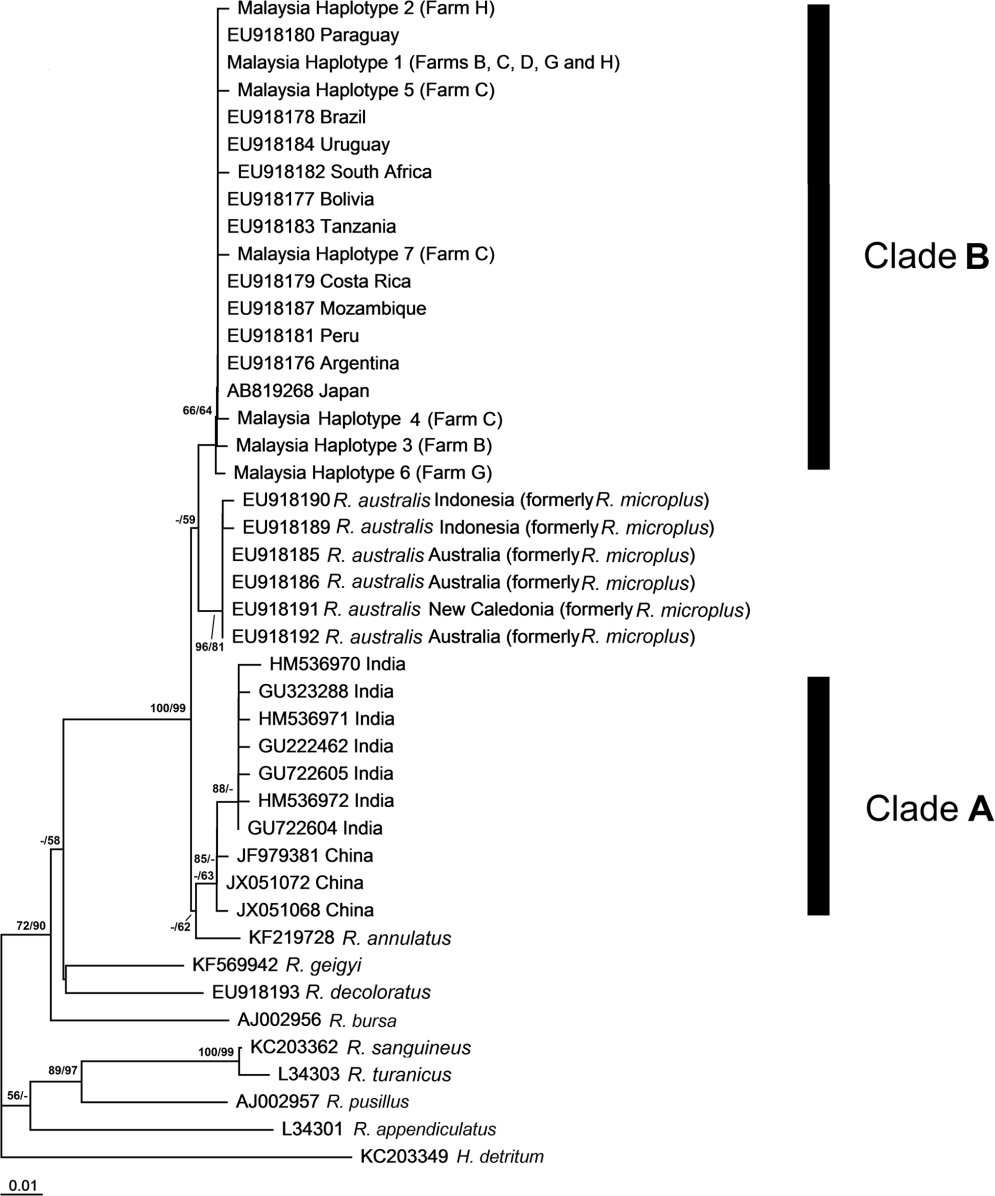
Neighbor-joining phylogenetic tree of *Rhipicephalus* taxa based on 16S rRNA sequences. Bootstrap values [neighbor-joining (NJ)/maximum likelihood (ML)] are shown on the branches

**Fig. 4:**
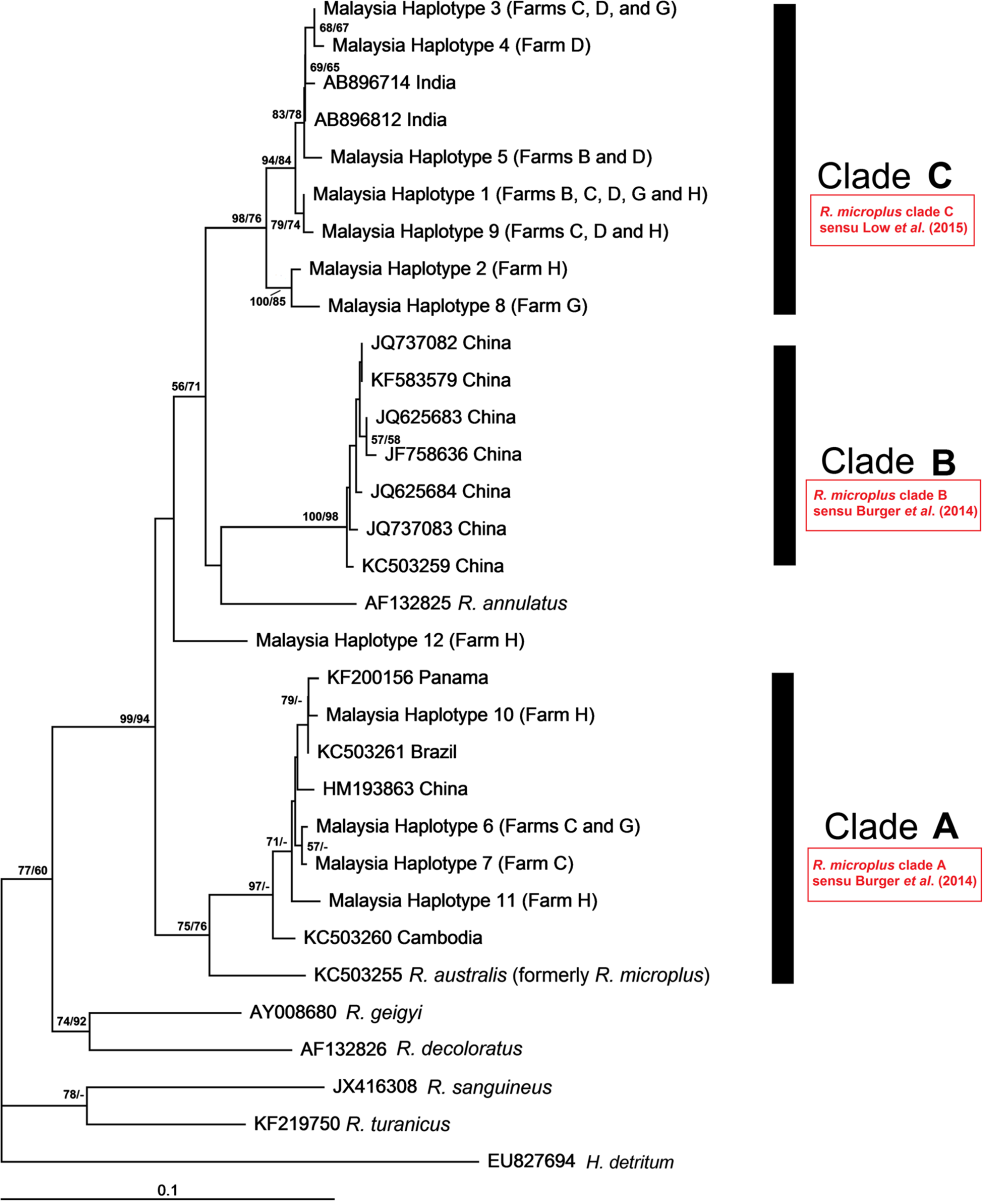
Neighbor-joining phylogenetic tree of *Rhipicephalus* taxa based on COI sequences. Bootstrap values [neighbor-joining (NJ)/maximum likelihood (ML)] are shown on the branches

NJ and ML analyses produced phylogenetic trees with the same topology but with different bootstrap support values (Figs. 2, 3 and 4). Only NJ trees were presented for the sequences of 16S rRNA and COI.

The 18 haplotypes (H1-H18) generated from the concatenated COI and 16S rRNA sequences of Malaysian *R. microplus* were subjected to phylogenetic analyses. *Rhipicephalus microplus* generally comprises four main clades: clade A (H4-H7), clade B (H14-H15), clade C (H16-H18) and clade D (H1-H2, H8-H13). There was no bootstrap support for haplotype H3, which formed a single clade (Fig. 2). Similar to the results of the haplotype network analysis, clade C is associated with ticks collected from the Nellore breed.

The 16S rRNA phylogenetic tree revealed two *R. microplus* genetic clades. One well-supported clade comprised *R. microplus* from China and India and was sister to *R. annulatus. Rhipicephalus microplus* from China was the basal member, forming a subclade with *R. microplus* from India. *Rhipicephalus australis* (formerly identified as *R. microplus*) from Australia, New Caledonia and Indonesia formed a well-supported clade. *Rhipicephalus microplus* from Malaysia, Japan, Africa (i.e., Mozambique, South Africa and Tanzania) and the Americas (i.e., Argentina, Bolivia, Brazil, Costa Rica, Paraquay, Peru and Uruguay) formed another clade (clade B) (Fig. 3).

The COI phylogenetic tree revealed three genetic clades of *R. microplus*. Clade A [*R. microplus* clade A sensu Burger *et al.* (2014)], the basal clade, comprised ticks from Southeast Asia (Cambodia and Malaysia), the Americas (Brazil and Panama) and China. Clade B [*R. microplus* clade B sensu Burger *et al.* (2014)], was restricted to ticks that originated from China. Clade C [*R. microplus* clade C sensu Low *et al.* (2015) comprised ticks from India and Malaysia. Haplotype 12 from Malaysia formed a distinct clade with no bootstrap support; thus, we excluded this clade as a valid genetic assemblage. *Rhipicephalus microplus* clade A sensu Burger *et al.* (2014) showed a sister relationship to *R. australis*. By contrast, *R. microplus* clade B sensu Burger *et al.* (2014) is more closely related to *R. annulatus*, with which they formed a sister relationship (Fig. 4).

## Discussion

In this study, we delineated the intraspecific genetic diversity and phylogeographical relationships of a major international pest of livestock, *R. microplus*, using 16S rRNA and COI markers. The results clearly indicate the advantages of using the COI gene for providing sufficient power to resolve the evolutionary relationships of *R. microplus*. We, like Burger *et al*. [10] found that the COI sequences were more variable and informative than 16S rRNA sequences. Exceptionally high levels of genetic variability were revealed by the COI gene. The intraspecific genetic distance or haplotype frequency reported here are notably higher than previously described for *R. appendiculatus*, *R. sanguineus* sensu lato, *R. guilhoni*, *R. pusillus*, *R. turanicus*, *R. muhsamae* and *R. bursa* [18, 34, 35]. Furthermore, the two, and perhaps three, genetic assemblages inferred from Malaysian *R. microplus* are more genetically diverse than those reported from other regions of the world (Fig. 4).

Among the five studied populations, *R. microplus* from Farm G was genetically diverse, as supported by the highest haplotype and nucleotide diversity based on COI, 16S rRNA and COI + 16S rRNA sequences (Table 1). As one of the farm management strategies in Malaysia, Farm G acts as the ‘weaner’s park’ and supplies weaned calves to other government farms (Farm A - Farm E and Farm G - Farm I) that are owned by the Department of Veterinary Services (DVS), Malaysia. Hence, we suggest that Farm G might be one of the sources for multiple introductions of *R. microplus* assemblages into other farms in Malaysia.

This study also identified distinct haplotype clusters as shown by a mixture of several genetically divergent groups in the Malaysian tick populations (Fig. 2). The significant genetic differentiation and high gene flow rates among the sampling sites support genetic admixture. The relatively low yet significant genetic differentiation and the widespread genetic admixture detected here were consistent with genetic studies of *R. microplus* populations from southern Texas [14]. This previous study proposed that southern Texas *R. microplus* has been introduced on multiple occasions, and these introductions have been associated with two main dispersal mechanisms (i.e., frequent short-distance dispersal and rare long-distance, human-mediated dispersal), thus promoting genetic admixture among tick populations. Additionally, the highly invasive and widespread movement of *R. microplus* [14, 36, 37] may facilitate its occurrence and observed high levels of gene flow. The studied cattle farms owned by the DVS in Malaysia serve as the sources of cattle production for imported and local breeds and eventually distribute the animals to all private farms throughout Malaysia. The natural dispersal ability of ticks might occur in parallel with human-mediated dispersal, causing genetic admixture and high gene flow in Malaysian populations.

Morever, this study also attempted to investigate whether haplotypes are distributed according to cattle breed. The specific association of haplotypes with other cattle breeds was not obvious except for some ticks collected from the Nellore breed which was imported from India. A distinct haplotype cluster (H16, H17, H18) was found for *R. microplus* ticks collected from these cattle. It is unknown whether this indicates a cause-effect association of haplotypes and this cattle breed. However, the prevalence of ticks has been typically associated with cattle breeds [38–40].

Over the last decade, a number of molecular approaches have been adopted to infer the phylogeographical relationships of *R. microplus* populations from different parts of the world [11–16]. As far as the molecular approaches are concerned, the mitochondria-encoded 16S rRNA and COI genes have invariably unraveled the distinct genetic assemblages of *R. microplus* and differentiated its closely related species, *R. annulatus* and *R. australis* [10, 15, 16]. Here, our 16S rRNA data were consistent with previous studies in which two 16S rRNA genetic clades were detected, with the first clade primarily confined to Africa/the Americas and the second clade confined to China/India. In contrast to the results found with the 16S rRNA gene, the COI phylogenetic data provided a new insight into the evolutionary lineages of *R. microplus* with the identification of three main assemblages: clade A sensu Burger *et al.* (2014) , comprising ticks from Southeast Asia, the Americas and China; clade B sensu Burger *et al.* (2014) , restricted to ticks that originated from China; and clade C sensu Low *et al.* (2015) , a new genetic assemblage discovered in this study, comprising ticks from India and Malaysia. Notably, the Chinese and Indian ticks were separated into different clades, in contrast to the 16S rRNA data. These results have unraveled the hidden diversity of Indian ticks, providing evidence that at least two distinct assemblages, namely *R microplus* clade A sensu Burger *et al.* (2014) and *R microplus* clade C sensu Low *et al.* (2015) exist in India. However, the majority of the COI haplotypes (seven out of 12) found in Malaysian ticks are shared with ticks from India. A plausible explanation for this could be because of the consequences of intense commercial cattle trade between both countries.

The comparative analysis in this study shows that *R. microplus* ticks represent at least three distinct genetic assemblages. Additional consideration should be given to the geographical differences between ticks in Malaysia and those from India, China and the Americas. Seasonal variations (e.g., temperature, climate and humidity) among the countries could be factors that shaped the observed patterns of genetic structure. Moreover, the role of anthropogenic disturbances in structuring the current genetic variability of *R. microplus* cannot be ruled out. This tick has been deemed the ‘world’s most pesticide-resistant tick’ and the evolution of biotypes with resistance to at least 44 active ingredients has been documented worldwide [41]. Hence, the ticks might have experienced selection pressure from pesticides leading to the observed high genetic variability. Additionally, because speciation might be driven by ecological divergence, the cryptic diversity of *R. microplus* might be underestimated. The recent study of Burger *et al*. [10] has suggested the presence of cryptic species in *R. microplus*, complicates the assessment of species status and is a common challenge in the Ixodidae. In this regard, crossbreeding studies from different isolates of *R. microplus*, including the new taxon *R. microplus* clade C sensu Low *et al*. (2015) discovered in the present study should be conducted in the near future.

## Conclusions

This study provides new insights into the distinct genetic assemblages in the tick *R. microplus*. We, therefore, conclude that the *R. microplus* complex consisting of at least five taxa: *R. australis*, *R. annulatus*, *R. microplus* clade A sensu Burger *et al.* (2014), *R. microplus* clade B sensu Burger *et al.* (2014) and the new taxon, *R. microplus* clade C sensu Low *et al.* (2015). The use of COI as the standard genetic marker in discerning the genetic assemblages of *R. microplus* from a broad range of biogeographical regions is proposed.

## Additional file

Additional file 1: Figure S1. Sequence alignment of *R. microplus* haplotypes (H1-H18) based on COI + 16S rRNA genes.
