# Supplementary figures and images for: Molecular characterisation of the tick Rhipicephalus microplus in Malaysia: new insights into the cryptic diversity and distinct genetic assemblages throughout the world
Source: Parasit Vectors. 2015 Jun 24;8:341. doi: 10.1186/s13071-015-0956-5 (PMC4482097; doi:10.1186/s13071-015-0956-5)

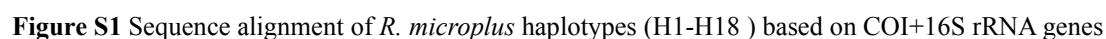

Supplement: Additional file 1: Figure S1. — Sequence alignment of R. microplus haplotypes (H1-H18) based on COI + 16S rRNA genes. [file 13071_2015_956_MOESM1_ESM.pdf]
